# Meeting Report: Aging Research and Drug Discovery

**DOI:** 10.18632/aging.203859

**Published:** 2022-01-28

**Authors:** Esther Meron, Maria Thaysen, Suzanne Angeli, Adam Antebi, Nir Barzilai, Joseph A. Baur, Simon Bekker-Jensen, Maria Birkisdottir, Evelyne Bischof, Jens Bruening, Anne Brunet, Abigail Buchwalter, Filipe Cabreiro, Shiqing Cai, Brian H. Chen, Maria Ermolaeva, Collin Y. Ewald, Luigi Ferrucci, Maria Carolina Florian, Kristen Fortney, Adam Freund, Anastasia Georgievskaya, Vadim N. Gladyshev, David Glass, Tyler Golato, Vera Gorbunova, Jan Hoejimakers, Riekelt H. Houtkooper, Sibylle Jager, Frank Jaksch, Georges Janssens, Martin Borch Jensen, Matt Kaeberlein, Gerard Karsenty, Peter de Keizer, Brian Kennedy, James L. Kirkland, Michael Kjaer, Guido Kroemer, Kai-Fu Lee, Jean-Marc Lemaitre, David Liaskos, Valter D. Longo, Yu-Xuan Lu, Michael R. MacArthur, Andrea B. Maier, Christina Manakanatas, Sarah J. Mitchell, Alexey Moskalev, Laura Niedernhofer, Ivan Ozerov, Linda Partridge, Emmanuelle Passegué, Michael A. Petr, James Peyer, Dina Radenkovic, Thomas A. Rando, Suresh Rattan, Christian G. Riedel, Lenhard Rudolph, Ruixue Ai, Manuel Serrano, Björn Schumacher, David A. Sinclair, Ryan Smith, Yousin Suh, Pam Taub, Alexandre Trapp, Anne-Ulrike Trendelenburg, Dario Riccardo Valenzano, Kris Verburgh, Eric Verdin, Jan Vijg, Rudi G.J. Westendorp, Alessandra Zonari, Daniela Bakula, Alex Zhavoronkov, Morten Scheibye-Knudsen

**Affiliations:** 1 Center for Healthy Aging, Department of Cellular and Molecular Medicine, University of Copenhagen, Copenhagen, Denmark; 2 Buck Institute for Research on Aging, Novato, CA 94945, USA; 3 Max Planck Institute for Biology of Ageing, Cologne, Germany; 4 Department of Genetics, Albert Einstein College of Medicine, Bronx, NY 10461, USA; 5 Institute for Aging Research, Department of Medicine, Albert Einstein College of Medicine, Bronx, NY 10461, USA; 6 Smilow Center for Translational Research, University of Pennsylvania, Philadelphia, PA 19104, USA; 7 Department of Molecular Genetics, Erasmus MC, Rotterdam, Netherlands; 8 Department of Neuroscience, Erasmus MC, Rotterdam, Netherlands; 9 Shanghai University of Medicine and Health Sciences, College of Clinical Medicine, Shanghai, China; 10 Max Planck Institute for Metabolism Research, Cologne, Germany; 11 Department of Genetics, Stanford School of Medicine, Stanford University, Stanford, CA 94305, USA; 12 Cardiovascular Research Institute, University of California, San Francisco, CA 94158, USA; 13 Institute of Clinical Sciences, Imperial College London, Hammersmith Hospital Campus, London W12 0NN, UK; 14 CECAD Research Center, Faculty of Medicine, University of Cologne, Cologne, Germany; 15 Institute of Neuroscience, Chinese Academy of Science, Shanghai, China; 16 FOXO Technologies Inc, Minneapolis, MN 55402, USA; 17 The Herbert Wertheim School of Public Health and Human Longevity Science, UC San Diego, La Jolla, CA 92093, USA; 18 Leibniz Institute on Aging, Jena, Germany; 19 Laboratory of Extracellular Matrix Regeneration, Institute of Translational Medicine, Department of Health Sciences and Technology, ETH Zürich, Schwerzenbach CH-8603, Switzerland; 20 Longitudinal Studies Section, Translational Gerontology Branch, National Institute on Aging, National Institutes of Health, Baltimore, MD 21224, USA; 21 Program for Regenerative Medicine, IDIBELL, Barcelona, Spain; 22 BioAge Labs, Inc., Richmond, CA 94804, USA; 23 Arda Therapeutics, San Carlos, CA 94070, USA; 24 HautAI OÜ, Tallinn, Estonia; 25 Division of Genetics, Department of Medicine, Brigham and Women’s Hospital, Harvard Medical School, Boston, MA 02115, USA; 26 Regeneron Pharmaceuticals, Inc., Tarrytown, NY 10591, USA; 27 Molecule, Schaffhausen, Switzerland; 28 Departments of Biology and Medicine, University of Rochester, Rochester, NY 14627, USA; 29 Department of Genetics, Erasmus MC, University Medical Center Rotterdam, Rotterdam, The Netherlands; 30 Laboratory Genetic Metabolic Diseases, Amsterdam UMC, University of Amsterdam, Amsterdam, The Netherlands; 31 L’Oréal Research and Innovation, Aulnay-sous-Bois, France; 32 ChromaDex Corp, Los Angeles, CA 90024, USA; 33 Gordian Biotechnology, San Francisco, CA 94107, USA; 34 Departments of Laboratory Medicine and Pathology, University of Washington, Seattle, WA 98195, USA; 35 Department of Genetics and Development, Columbia University Medical Center, New York, NY 10032, USA; 36 Department of Molecular Cancer Research, Center for Molecular Medicine, Division of Biomedical Genetics, University Medical Center Utrecht, Utrecht University, Utrecht, The Netherlands; 37 Departments of Biochemistry and Physiology, Yong Loo Lin School of Medicine, National University Singapore, Singapore; 38 Center for Healthy Longevity, National University Health System, Singapore; 39 Division of General Internal Medicine, Department of Internal Medicine, Mayo Clinic, Rochester, MN 55905, USA; 40 Department of Clinical Medicine, University of Copenhagen, Copenhagen, Denmark; 41 Centre de Recherche des Cordeliers, Université de Paris, Sorbonne Université, Inserm U1138, Paris, France; 42 Sinovation Ventures and Sinovation AI Institute, Beijing, China; 43 Institute for Regenerative Medicine and Biotherapies, INSERM UMR 1183, Montpellier, France; 44 Nagi Bioscience, Ecublens, Switzerland; 45 USC Davis School of Gerontology, University of Southern California, Los Angeles, CA 90089, USA; 46 Department of Health Sciences and Technology, ETH Zurich, Zurich, Switzerland; 47 Department of Human Movement Sciences, @AgeAmsterdam, Faculty of Behavioural and Movement Sciences, Amsterdam Movement Sciences, Vrije Universiteit Amsterdam, Amsterdam, The Netherlands; 48 Department of Medicine, Yong Loo Lin School of Medicine, National University Singapore, Singapore; 49 Max Perutz Labs, The University of Vienna, Vienna, Austria; 50 Institute of Biology of FRC Komi Science Center of Ural Division of RAS, Syktyvkar, Russia; 51 Russian Clinical and Research Center of Gerontology, Moscow, Russia; 52 Institute on the Biology of Aging and Metabolism, Department of Biochemistry, Molecular Biology and Biophysics, University of Minnesota, Minneapolis, MN 55455, USA; 53 Insilico Medicine, Hong Kong Science and Technology Park, Hong Kong; 54 Columbia Stem Cell Initiative, Columbia University, New York, NY 10032, USA; 55 Tracked.bio, Copenhagen, Denmark; 56 Cambrian Biopharma, New York, NY 11205, USA; 57 Hooke London by Health and Longevity Optimisation, London, UK; 58 Department of Neurology and Neurological Sciences and Paul F. Glenn Center for Biology of Aging, Stanford University School of Medicine, Stanford, CA 94305, USA; 59 Department of Molecular Biology and Genetics, Aarhus University, Aarhus, Denmark; 60 Department of Biosciences and Nutrition, Karolinska Institute, Stockholm, Sweden; 61 Department of Clinical Molecular Biology | UiO, University of Oslo and Akershus University Hospital, Norway; 62 Institute for Research in Biomedicine (IRB Barcelona), Barcelona Institute of Science and Technology (BIST), Catalan Institution for Research and Advanced Studies (ICREA), Barcelona, Spain; 63 Blavatnik Institute, Department of Genetics, Paul F. Glenn Center for Biology of Aging Research at Harvard Medical School, Boston, MA 94107, USA; 64 TruDiagnostic, Lexington, KY 40503, USA; 65 Departments of Obstetrics and Gynecology, Genetics and Development, Columbia University, New York, NY 10027, USA; 66 Division of Cardiovascular Medicine, University of California, San Diego, CA 92093, USA; 67 Novartis Institutes for Biomedical Research, Cambridge, MA 02139, USA; 68 Longevity Vision Fund, New York, NY 10022, USA; 69 Department of Public Health, University of Copenhagen, Copenhagen, Denmark; 70 OneSkin, San Francisco, CA 94107, USA

**Keywords:** aging, drug discovery, conference, AI, longevity

## Abstract

Aging is the single largest risk factor for most chronic diseases, and thus possesses large socioeconomic interest to continuously aging societies. Consequently, the field of aging research is expanding alongside a growing focus from the industry and investors in aging research. This year’s 8th Annual Aging Research and Drug Discovery (ARDD) meeting was organized as a hybrid meeting from August 30th to September 3rd 2021 with more than 130 attendees participating on-site at the Ceremonial Hall at University of Copenhagen, Denmark, and 1800 engaging online. The conference comprised of presentations from 75 speakers focusing on new research in topics including mechanisms of aging and how these can be modulated as well as the use of AI and new standards of practices within aging research. This year, a longevity workshop was included to build stronger connections with the clinical community.

## INTRODUCTION

As the world population ages, the need for a deeper understanding of healthy aging is becoming increasingly important. In recent years, the Aging Research and Drug Discovery (ARDD) meeting has brought experts of different sectors together, thereby providing researchers and representatives from the industry a platform to exchange ideas, thoughts and expertise and to foster collaborations. This year the 8th ARDD conference, organized by Morten Scheibye-Knudsen, University of Copenhagen, Alex Zhavoronkov, Insilico Medicine and Daniela Bakula, University of Copenhagen, was held in Copenhagen, Denmark from August 30th to September 3rd 2021. The meeting featured presentations from 75 world leading aging experts with participation of over 2000 attendees, attending either in person or virtually, as well as a Longevity Medicine Workshop and a panel session of venture capitalists. As a new initiative, the Inspire Longevity program was started this year, aiming to inspire young students to engage in longevity research.

This report covers the many exciting research areas presented at the conference, ranging from cellular maintenance pathways and how they can be modulated to promote healthy aging, new thoughts on stem cell rejuvenation and senotherapeutics, to new model systems in aging. It explores the many advances in AI and aging clocks, and the innovations in drug discovery from preclinical to clinical studies.

### Age-dependent control of cellular maintenance processes

Understanding cellular maintenance processes such as the regulation of genome stability and proteostasis is an important area of aging research [1]. With age there is an accumulation of somatic mutations known as somatic mosaicism, creating a mutational burden that can lead to decreased transcriptional stability. Jan Vijg, Albert Einstein College of Medicine, USA, presented new advances in single cell sequencing and multi-omics that allows the study of de novo mutations leading to somatic mosaicism [2]. Björn Schumacher, University of Cologne, Germany, highlighted DNA damage as a main driver of aging. Expanding on the crosstalk between germline and somatic cell maintenance, he showed that DNA damage induced H3K4me2 in *C. elegans* promotes recovery of protein biosynthesis and homeostasis thereby linking DNA repair and protein homeostasis [3]. Notably, there is also a correlation between protein fidelity and lifespan in different organisms. Filipe Cabreiro, Imperial College London, UK, and University of Cologne, Germany, discovered the ribosomal protein S23 (RPS23) K60R mutation to be a hyperaccuracy mutant. RPS23 K60R mutation leads to improved translation accuracy, heat resistance, and longer lifespan in *S. pombe, C. elegans* and *D. melanogaster* without reducing translation, making translational fidelity a potential therapeutic target [4]. Notably, protein stability varies across tissues with age independently of cell proliferation rate, which suggests shared and unique aging vulnerabilities of proteins across tissues, as presented by Abigail Buchwalter, University of California, USA [5].

### Longevity pathways

Cellular pathways associated with increased longevity have been extensively studied for many years. Consequently, drugs targeting these pathways have been shown to have a positive effect on lifespan in different model organisms.

Rapamycin is known to increase the lifespan of organisms by acting through mTOR. Linda Partridge, Max Planck Institute for Biology of Ageing, Germany, highlighted the importance of intestinal homeostasis and aging. Their research revealed that short term and early treatment with rapamycin extends lifespan in *D. melanogaster* as much as chronic rapamycin treatment. The short-term rapamycin treatment induced long-term increase in autophagy, decrease in pathology of fly intestine. Similarly, short term rapamycin treatment in mice caused a reduction in intestinal pathology for up to 6 months after rapamycin treatment (unpublished data). Moreover, there is an unconventional intestine sex-specific TORC1-histone axis which uncovers a new aspect to the improved longevity with rapamycin as shown by Yu-Xuan Lu from Max Planck Institute for Biology of Ageing, Germany. The observed sex differences and possible other unconventional pathways need to be considered prior to rapamycin treatment [6].

Another well studied pathway in aging is nicotinamide adenine dinucleotide (NAD) metabolism and the age-dependent NAD decline. Joseph A. Baur, University of Pennsylvania, USA, showed that the mitochondrial protein SLC25A51 is a transporter that modulates the transport of NAD into the mitochondria. An AI-based drug screen revealed promising results that this transporter can be modulated and in the future be a novel way of preventing age-dependent NAD decline [7]. Traditionally, the NAD pathway has been modulated by supplementing with NAD precursors such as nicotinamide riboside (NR). Frank Jaksch from ChromaDex Corp. suggested that NR supplementation could be a potential treatment for orphan diseases characterized by DNA damage and mitochondrial dysfunction which has been demonstrated in preclinical models. Currently, clinical studies are examining the potential effects of NR supplementation [8, 9]. However, Riekelt Houtkooper’s group, Amsterdam UMC, Netherlands, point to NAD+ precursors having shown limited translatability in human trials and has instead characterized a novel reduced NAD+ precursor, NMNH. NMNH increases NAD+ levels to a greater extent than traditional precursors NR and NMN and sustains elevated NAD+ in mice [10].

Brain aging is associated with behavioral deterioration and may be improved by enhancing levels of neurotransmitters. Shiqing Cai, Institute of Neuroscience, China, utilized a genome-wide RNAi screen in *C. elegans* and found that overexpression of the two epigenetic regulators BAZ2B and EHMT1 may cause decreased levels of serotonin and dopamine and decreased mitochondrial function. Further, the gene expression of both positively correlates with the progression of Alzheimer’s disease and are therefore potential novel anti-aging target genes [11]. Another promising anti-aging target is the mitochondrial permeability transition pore (mPTP), which increases with age and age-related diseases such as Alzheimer’s disease, Parkinson’s disease, and ischemia/reperfusion injuries. OSCP, a subunit of F-ATP synthase, is an important regulator of the mPTP and loss of OSCP leads to a decreased lifespan in *C. elegans* and initiation of a maladaptive mitochondrial unfolded protein response (UPR^mt^). Thus, mPTP/UPR^mt^ may be a contributor of aging and age-related disease as shown by Suzanne Angeli, Buck Institute for Research on Aging, USA [12].

Collin Ewald from ETH Zurich, Switzerland, proposed extracellular matrix homeostasis as a novel longevity pathway that is highly conserved among species. Supplements with components of the extracellular matrix which are well known and safe could therefore be used as a potential longevity pill [13]. Looking at hydrogen sulfide (HSO) homeostasis, Alexey Moskalev, Russian academy of Sciences, Russia, has found that its disruption is associated with aging and therefore a potential gero-therapeutic target. He showed that combined genetic and therapeutic interventions of HSO metabolism led to beneficial effects of HSO production and life- and health span extension in *D. melanogaster* along with stress resistance [14]. Another novel mechanism to regulate homeostasis at the embryonic level is through adrenal steroidogenesis. Gerard Karsenty, Columbia University, USA, showed that embryonic osteocalcin determines adrenal development. As a result, embryonic but not postnatal osteocalcin is essential for life-long maintenance of whole-organism homeostasis through its regulation of blood pressure and electrolyte metabolism. In this capacity embryonic osteocalcin determines healthy aging [15]. Adam Antebi, Max Planck Institute for Biology of Ageing, Germany, presented their latest study in which they used metabolic profiling of long-lived *C. elegans* strains and identified the folate/ methionine cycle as a convergence point of aging pathways. This elucidates how single genes or metabolites can regulate lifespan through multiple pathways [16].

With the high correlation between age and the risk of COVID-19 related deaths, novel research is emerging in aging pathways. Activin signalling has been seen to increase with age, and is especially elevated in acute respiratory disease syndrome (ARDS) patients and with relevance to COVID-19. David Glass, Regeneron, USA showed that Activin A production can be stimulated by a cytokine storm and is seen in most severe COVID-19 patients. Activin A may therefore be a biomarker of increased risk of ARDS in COVID-19 patients. However, Activin A is also associated with decreased viral load in cell culture, and is therefore a problematic therapeutic target [17].

### Cellular stress and aging

Looking at aging as the progressive loss of health treatment should be focused on drugs *for* health and not *against* disease, stated Suresh Rattan, Aarhus University, Denmark. In this context, he highlighted the biological response hormesis and how stress induction can upregulate adaptive responses and how hormetins in various forms promote general health [18]. Michael Kjær, University of Copenhagen, Denmark, elaborated on a physical hormetin, exercise. A study of his group revealed that life-long endurance training causes an increased muscle mass maintenance and strength in older individuals. The improved muscle function is possibly evoked by an increased muscle growth and anti-inflammatory effects of physical training. Notably, these muscle improvements can be achieved even when the training is started in old age, though the extent of improvement diminishes with age [19]. During different stress conditions such as endurance training, the cells adapt through different signalling pathways. One signaling pathway is the MAP kinase signalling cascade, and here Simon Bekker-Jensen, University of Copenhagen, Denmark, showed that the two MAP3K isoforms ZAKa and ZAKb respond to stress and promote adaptive mechanisms. Lack of ZAKa leads to a shorter lifespan in *C. elegans* in response to amino acid starvation. ZAKb responds to mechanical stress and its deletion in humans is associated with severely impaired muscle function [20] (unpublished).

### The benefits of dietary restriction

Another well-described stressor that has been shown to extend lifespan as well as improve healthspan is dietary restriction (DR). With age there is a decrease in RNA but increase in RNA polymerase II (RNAPII), specifically elongation RNAPII, suggesting lower RNA productivity of RNAPII with age as presented by Jan Hoeijmakers, Erasmus Medical Center Rotterdam, Netherlands. This preferentially affects long genes being suppressed in expression leading to an imbalanced RNA pool. DR compensates for this effect by reducing DNA damage load and alleviating transcription stress (unpublished). Maria Birkisdottir, Erasmus MC, Netherlands, showed that DR but not rapamycin leads to an increased health- and lifespan in ERCC1^−/Δ^ mice. Similarly, DR but not rapamycin prevents the observed degeneration of Purkinje neurons in ERCC1^−/Δ^ mice, indicating that DR and rapamycin differ in their mode of action [21]. Moreover, fasting or a ketogenic diet have been shown by Thomas A. Rando Stanford, USA to cause the muscle stem cells to enter a deep quiescent state mediated through HDAC1 and p53. This causes an increased resilience of aged muscle stem cells [22].

The beneficial effects of DR are lost in late-life interventions, potentially because the induction of mitochondrial function is necessary for the response but declines with age. Lenhard Rudolph, Leibniz Institute on Aging, Germany, showed new insides into new mechanisms that can rescue the capacity of late life DR (administered in old animals) to induce health promoting stress signals and improvements in stem cell function and lifespan (unpublished). Additionally, Maria Ermolaeva, Leibniz Institute on Aging, Germany, discussed how the protective effects of metformin on lifespan are abrogated in late-life interventions in *C. elegans*, which is likely caused by mitochondrial dysfunction associated with age. Increased mitochondrial content or ATP supplementation rescues the aging effect on metformin toxicity and restores its beneficial effects on healthy aging [23].

New findings have been made in dietary restriction mimetics as an easier therapeutic option. Sarah J. Mitchell, ETH Zürich, Switzerland, presented an alternative late-life intervention in 21 month old mice with the fumagillin derivative, ZGN1062, a methionine aminopeptidase 2 inhibitor, which showed positive effects on lifespan and healthspan, even more so than late-life calorie or methionine restriction. This suggested that the drug could be an alternative to calorie restriction (unpublished). Michael R. MacArthur, ETH Zurich, Switzerland, elaborated on their findings regarding ZGN1062. It rapidly decreases food-intake through activation of p53 signaling, leading to down-stream induction of GDF15, and reduced food intake (unpublished). Guido Kroemer, Université de Paris, France, pointed out that caloric restriction mimetics enhances autophagy which improves longevity. Additionally, caloric restriction mimetics can be used in the treatment of diseases such as cancer; here the Kroemer group showed that the IGF1R inhibitor picropodophyllin enhance the efficacy of chemoimmunotherapy by stimulating immunosurveillance in mice [24, 25]. Valter Longo, USC Davis School of Gerontology, USA, discussed fasting mimicking diets in relation to vitamin C treatment of cancer cells. Vitamin C kills KRAS mutant cancer cells, but the effect is reduced by vitamin C activation of heme oxygenase 1 (HO-1) that induces ferritin which scavenges free Fe^2+^. This prevents the reaction of Fe^2+^ with H_2_O_2_ to produce hydroxy radicals. However, combined with a fasting mimicking diet the effects of vitamin C on HO-1 are inhibited, leading to increased levels of hydroxy radicals, increasing the efficiency of this treatment against cancer cells [26].

Pam Taub, University of California San Diego School of Medicine, USA, presented another alternative to DR. Time restricted eating is the alignment of eating patterns to the circadian rhythm, with a 8–10 hour daytime feeding window. This causes a metabolic shift from glucose to ketones leading to a low-grade state of ketosis which helps to build metabolic resilience, improves endothelial function and reduces inflammation. This metabolic resilience is beneficial against metabolic syndrome, and potentially also Covid-19 which shows similar hallmarks [27].

Further elucidating on the nutrient signaling pathways, Jens Brüning, Max Planck Institute for Metabolism Research, Germany, presented on the POMC and AgRP nutrient sensing neurons in the arcuate nucleus. The orexigenic AgRP is inhibited by insulin, and insulin binding to AgRP is necessary for normal insulin suppression of hepatic glucose production [28]. He further showed that insulin receptors on tanycytes are required for insulin transport to the CNS and actions in the arcuate nucleus (unpublished). Furthermore, Christian Riedel, Karolinska Institute, Sweden, showed that under reduced insulin/insulin-like growth factor signaling (ISS) in *C. elegans*, chromatin undergoes substantial accessibility changes. These promote activity of the transcription factor LIN-39 in neurons. It is proposed that LIN-39 is required for the formation or maintenance of neurons required for the longevity seen under reduced ISS (unpublished).

### Unlocking stem cell rejuvenation

One characteristic of aging is stem cell exhaustion. Thus, understanding the properties and maintenance of stem cells may elucidate how to slow down the aging process. Maria Carolina Florian, Program for Regenerative Medicine, IDIBELL, Barcelona, Spain, discussed how aged hematopoietic stem cells (HSCs) localize in clusters away from the endosteum. Only functional aged HSCs are located at sinusoids, thus, the sinusoids are critical for their support, which is especially important to consider when treating with chemotherapy as it disrupts the sinusoids [29]. However, unlike other tissue, old HSCs are refractory to systemic rejuvenation interventions, as found by Emmannuella Passegué, Columbia University, USA. Old HSCs are not affected by exposure to young blood cells or known longevity interventions such as DR, and young HSCs are likewise not affected by old HSCs. This suggests that the focus should be on interventions to delay aging rather than rely on rejuvenation HSCs [30].

Alternatively, induction of pluripotent stem cells has been shown to rejuvenate tissue. Jean-Marc Lemaitre, Institute of Regenerative Medicine and Biotherapies of Montpellier, France, presented that even a short transient induction of the Yamanaka factors in a mouse model of premature aging early in life improved body composition and fitness throughout life as well as attenuated some of the aging related changes observed in the different tissues. The proposed mechanism is through epigenetic reprogramming in multiple organs [31]. On this topic, David A. Sinclair, Harvard Medical School, USA, showed that aging-driven epigenetic and gene expression changes in the central nervous system can be safely reversed to restore vision by inducible adeno-associated viruses expressing polycistronic Oct4, Sox2 and Kif4, and that the effect was dependent on DNA demethylation [32]. Furthermore, rejuvenation can be obtained *in vivo* with partial reprogramming when Yamanaka factors are expressed for one week followed by a two-week recovery, as explained by Manuel Serrano, IRB Barcelona, Spain. However, the Serrano group is currently investigating how to rejuvenate independently of the Yamanaka factors. In addition, Serrano explained how repeated cycles of expression of the Yamanaka factors prevent the gradual loss of hippocampal neurogenesis associated to aging, and thereby it prevents the loss of memory performance of old mice. [33].

### Senolytics as an aging therapeutic

Senescence is a known feature of aging, thus, the consequences of senescence as well as the use of senolytics as potential treatments are extensively studied. James Kirkland, Mayo Clinic, USA, described that transplanting of senescent cells in young mice caused spread of senescence to other cells and organs and led to increased frailty. The co-treatment of the senolytics dasatinib and quercetin broadly inhibited senescent cell anti-apoptotic pathways in mice, decreasing the number of naturally occurring senescence-associated secretory phenotype (SASP) presenting cells [34].

Furthering the discussion on SASP, tissue-specific knockout of the DNA repair endonuclease ERCC1-XPF revealed that the hematopoietic system, specifically, is a systemic driver of secondary senescence in a cell non-autonomous way through SASP, causing senescence to be found in non-immune cells in numerous organs, as presented by Laura Niedernhofer, University of Minnesota, USA. This makes senescent immune cells a good target for senolytics, such as fisetin, as well as potentially a good biomarker of tissue damage and multi-morbidities. She further showed that senescent cells in old mice cause an over-reaction to PAMPs, driving a cytokine storm that drives organ failure and death upon infection with a pathogen such as β-coronaviruses, an effect that can be ameliorated by treatment with the senolytic fisetin [35, 36]. Eric Verdin from the Buck Institute, USA, showed how SASP promotes macrophage proliferation and macrophage senescence with age. This leads to an M1 switch which is key for macrophages involved in inflammaging. Macrophages should thus be considered as a potential therapeutic target [37]. The deleterious effects of SASP are seen across multiple tissues. Christina Manakanatas, University of Vienna, Austria, specifically explained how progerin expressing endothelial cells initiate a senescence signaling cascade mediate through the p53/p21 axis and miR34. This results in secretion of SASP and miR34 mediating paracrine signaling which could explain the cardiovascular diseases seen in HGPS (unpublished).

The potential of senotherapeutics is also emerging within the field of skin aging. The CSO of OneSkin Alessandra Zonari presented their latest findings regarding Peptide 14, identified through a cell-based screening. Treatment of aged skin with Peptide 14 caused a reduction in senescence and inflammatory markers and led to an increase in skin health with higher efficiency than rapamycin treatment [38]. Sibylle Jäger, L’Oreal Research and Innovation, presented a novel salicylic acid derivative C8-SA is known to promote longevity in *C. elegans* and prevent oxidative damage in humans by stimulating endogenous antioxidant defenses in an AMPK DAF-16 dependent manner (unpublished, L’Oreal).

From the point of senescent cells, Peter de Keizer, University Medical Center Utrecht, the Netherlands, stressed that senescence is heterogeneous and showed that specific subsets of senescent cells can be defined using imaging-mass cytometry (IMC), as well as real-time LMNB1-GFP reporter-based sorting and single cell RNA-seq. This, he argues, is crucial in the treatment of senescent cells. He showed their improved FOXO4-p53 DRI peptides have specificity for such a subset of senescent cells, called “scarred” cells – with striking effects in models for metastatic colon cancer and triple negative breast cancer [39].

### Looking at diverse models of aging

As aging is multifaceted, using segmental models of aging can give us insight into different aspects of the many mechanisms of aging. A model system of aging can be an individual organ, other organisms, or a human disorder mimicking part of the aging process.

Brian Kennedy, National University of Singapore, Singapore, showed how Alzheimer’s disease can be used as a model of neuronal aging. Increased lamin A expression is seen in aging and in Alzheimer’s disease. ApoE KO in neuronal stem cells causes an increase in Lamin A and decrease in Lamin B. In astrocytes, this lead to accelerated senescence and increased inflammation which may affect neurons through an increase in inflammatory and senescence markers (unpublished).

Moving on to model organisms, Dario Valenzano, Max Planck Institute for Biology of Aging and Leibniz Institute on Aging, Germany, discussed the use of African turquoise killifish as a model of aging. Killifish show a diverse microbiome across different habitats and age, and their microbiome can be used as a readout of the health of the host. Treating killifish with the microbiome of a young fish has been shown to modulate lifespan [40, 41]. Vera Gorbunova, University of Rochester, USA, studies long lived mammals such as the bowhead whale and bats and has shown a strong link between longevity and genome maintenance, and specifically upregulation of double-stranded break (DSB) repair. While bowhead whales exhibit replicative senescence, they have attenuated SASP response and reduced inflammation. Additionally, their DSB repair efficiency is higher compared to humans (unpublished). Similarly, the little brown bat shows a high NHEJ efficiency compared to rats and a dampened inflammatory response [42, 43].

Daniela Bakula, University of Copenhagen, Denmark, presented how to use machine learning tools including aging clocks to identify diseases that show accelerated aging features. In this work they characterized a novel premature aging syndrome and investigated the underlying cellular function causing the observed accelerated aging pace (unpublished data).

### Tick, tock, aging clocks and biomarkers of aging

A rapidly emerging method to study the aging process are aging clocks. Clocks can be trained on datasets extending from single cell -omics, movement data and blood parameters, to survey-based data and psychological factors. Anne Brunet, Stanford, USA, presented a single-cell RNA-seq based clock that can accurately predict chronological age across the neural stem cell niche and is very cell type specific. The clock is driven by cell-specific genes and it can be used to test rejuvenation interventions (unpublished). Furthermore, Alex Trapp, Harvard Medical School, USA, introduced the first single cell epigenetic clock that allows the aging process to be dissected in specific cell types. Additionally, an extension of the method called shallow sequencing decreased the sequencing cost compared to current deep epigenetic sequencing, while maintaining the accuracy [44]. Vadim Gladyshev, Harvard Medical School, USA, used multi-tissue epigenetic and aging clocks to show rejuvenation during early embryogenesis in mice and humans. He proposed a model wherein the germline ages during development and through adulthood but rejuvenates in the offspring after conception and reverts to the point of ‘ground zero’, which marks the beginning of aging [45, 46].

Georges Janssens, Amsterdam UMC, Netherlands, discussed the use of population data to create clocks. They created a movement clock using population data that predicts biological age. Additionally, the clock can be used to find factors such as diets or drugs associated with healthier aging (unpublished). Alex Zhavoronkov, Insilico Medicine, Hong Kong, presented the many aging clocks developed by Deep Longevity as well as their newest clock, MindAge, which is a psychological aging clock. MindAge is trained on survey-based data with the purpose of being used in clinics, by insurance companies, and by employers [47]. Kai-Fu Lee, CEO Sinovation Ventures, Beijing, China also advocated the use of aging clocks in the clinics, however, he emphasized the need for more specific clocks trained on better data-sets and combining multiple clocks. Many clocks such as GlycanAge, PhotoAge and BloodAge are used in the clinic in order to predict the biological age of the patient, as explained by Dina Radenkovic, Hooke London by Health and Longevity Optimisation, UK. Despite a believed high predictive value, guidelines should be created on how to apply the clocks to a clinical setting [48].

TruDiagnostic has developed a clock that predicts the pace of aging rather than true biological age as shown by Ryan Smith. DunedinPACE is a blood DNA methylation clock showing pace of aging targeted for individual persons [49].

Aging clocks are a key factor in order to assess human health and identifying promising interventions. However, Brian Chen from FOXO Technologies, USA stressed the importance of validation and systematic characterization of described clocks – like all other biomarkers – will be essential to drive the aging industry forward [50].

The evaluation of the efficacy of aging interventions requires a combination of different molecular biomarkers. A specific population of interest in the study of biomarkers are centenarians, as studied by Yousin Suh, Columbia University, USA. She has examined centenarian gene variants in conserved pathways of aging to find alleles that promote longevity such as the longevity-associated variant of SMAD3-gene, which causes decreased expression of SMAD3 thereby preventing the effects on lifespan and senescence (unpublished).

An additional approach to discover biomarkers was presented by Anastasia Georgievskaya, Haut.AI, Estonia. Using AI to discover visual, facial biomarkers that are gender and age specific can be obtained from a selfie. These biomarkers can potentially be used to measure the effect of longevity interventions in a clinical setting [51]. Rudi Westendorp, University of Copenhagen, Denmark, proposed that our current biomarkers change with age and have only little predictive value for clinical prediction. His group has developed a recurrent neural network to predict frailty and subsequent need for preventative services in old persons which showed better predictive value compared to established methods [52]. An alternative approach, using dynamic indicators of the response to stressors seem to provide more information on resilience of organisms (unpublished).

### Rethinking aging research: from preclinical to clinical

Effective aging therapeutics for humans are dependent on the pipeline from drug discovery to testing in model organisms and through to human clinical trials. By advancing on how we approach drug discovery and the transition from preclinical to clinical trials, we can expect to see cheaper, faster, and more effective therapeutics emerge.

The use of AI has led to broader and less biased approaches for the discovery of potential therapeutics. Ivan Ozerov, Insilico Medicine, Hong Kong, presented PandOmics as a platform that uses an AI based-approach to discover new therapeutic targets for different diseases and disease subtypes. This can ease and speed up the discovery of potential drug candidates [53]. Morten Scheibye-Knudsen, University of Copenhagen, Denmark further highlighted the importance of AI-based approaches in finding lifespan-improving drugs. Using Danish population-based prescription data, the Scheibye-Knudsen lab has identified drugs associated with longer lifespan. Additionally, by using *in silico*-based high-throughput screening, potential therapeutics that stimulate age-associated DNA repair pathways have been characterized (unpublished). Alice Ruixue Ai, University of Oslo and Akershus University Hospital, Norway, showed an AI-based approach to identify potent mitophagy inducers. The two lead compounds diminished Tau pathology and improved the memory of an Alzheimer’s disease mouse model, thus, highlighting the potential of use of AI in a preclinical setting (unpublished).

Optimizing and upscaling methods of preclinical testing will allow faster and cheaper drug discovery. Gordian Biotechnology, USA, created a setup that simultaneously tests the *in vivo* efficacy of multiple drugs prior to clinical testing using pooled *in vivo* screening, as presented by Martin Borch Jensen. He highlights this as a way to increase the likelihood of a drug succeeding through clinical trials [54]. Calico has developed a semi-automated platform for measuring healthspan in mice and it can be used to detect several aging phenotypes, easing the labor-intensive and time consuming testing of longevity drugs in mice, as shown by Adam Freund [55].

Matt Kaeberlein, The University of Washington, USA, presented their new WormBot, a *set it and forget it* method of large-scale intervention testing in *C. elegans*. He stressed the importance of broad and unbiased screening of intervention beyond known pathways and in different combinations [56]. Additionally, a high throughput, fully automated *C. elegans* organism-on-chip method that can be used for lead optimization, early toxicology and drug discovery was described by David Liaskos, The École Polytechnique Fédérale de Lausanne (EPFL), Switzerland. Development, reproduction, motility and survival information can be extracted resulting in faster preclinical testing [57]. Michael Petr Tracked.Bio, presented their use of AI-based software to track motion data of drosophila and mice, which can be used to study movement as features of healthspan as well as survival data [58].

The transition from preclinical to clinical studies remains a challenging and protracted process. Andrea Maier, National University of Singapore, Singapore, stressed the need for an unbiased standardized characterization and reporting of research outcome in the form of a minimum required dataset and trial networks to combat the poor transition currently seen. Additionally, she suggested drug repurposing as a relatively cheap and faster way for new age-related treatments [59, 60]. The great benefit of drug repurposing was also highlighted by Nir Barzilai, Albert Einstein College of Medicine, USA, who studies the repurposing of FDA approved drugs such as metformin and canagliflozin [61]. As aging is not currently recognized as a disease by the FDA, drug repurposing is an invaluable tool [62]. Anne-Ulrike Trendelenburg, Novartis Institutes for Biomedical Research, USA, presented a roadmap on how to conduct preclinical testing of geroprotectors for easy clinical translation. She highlighted biomarkers for frailty and targeting multi morbidity as crucial points to consider during preclinical testing of gerotherapeutics [63, 64].

Furthering the discussion on new common standards of studies, Luigi Ferrucci, NIA-NIH, USA, presented the value of using longitudinal perspectives in population studies. Longitudinal studies are more precise for measuring biomarkers and factors of aging, and looking specifically at longitudinal changes and trajectories in aging allows the study of functions of aging across lifespan [65].

### Longevity industry landscape

This year at the conference, multiple companies presented their way of tackling drug discovery. James Peyer, CEO of Cambrian biopharma, a Distributed Development Company, discussed their strategy to use established regulatory frameworks in order to develop new healthspan extending medicines. They have established a pipeline of new drugs that target causes of aging and age-related diseases and have candidates for clinical testing [66]. Furthermore, the CEO of BioAge, Kristen Fortney presented their pipeline that starts with analyzing human longitudinal data and ends with clinical testing of a validated compound to discover treatments that can extend healthy lifespan by targeting the molecular causes of aging. This has currently led to three compounds in clinical trials [67].

Moreover, new ways of funding are emerging such as VitaDAO, a new decentralized collective funding of early-stage longevity research as presented by co-founder Tylor Golato. Their goal is to extend human lifespan by researching, financing, and commercializing longevity therapeutics in an open and democratic manner [68]. Also, among venture capitalists there is an increasing awareness of the aging field. Kris Verburgh from the Longevity Vision Fund presented their work in investing in technologies to extend lifespan and improve healthspan [69].

## CONCLUSION

Aging is a multifaceted process, making research complex and diverse in topics. This year’s conference has elucidated the latest findings of aging mechanisms using known and novel models of aging and the modulation of these and understanding how to slow aging by stem cell rejuvenation or senotherapeutics. Finally, the latest advances in AI were presented as well as new ideas on how to facilitate the transition from preclinical to clinical studies. This effort is shared by an emerging industry involved in every step of the research journey making the prospect of healthy aging very real. The future is bright.
